# Evolutionary perspective on mammalian inorganic polyphosphate (polyP) biology

**DOI:** 10.1042/BST20230483

**Published:** 2023-10-16

**Authors:** Filipy Borghi, Adolfo Saiardi

**Affiliations:** Laboratory for Molecular Cell Biology, University College London, London WC1E 6BT, U.K.

**Keywords:** evolution, inorganic polyphosphate, inositol pyrophosphate, metabolism, mitochondria, nucleus

## Abstract

Inorganic polyphosphate (polyP), the polymeric form of phosphate, is attracting ever-growing attention due to the many functions it appears to perform within mammalian cells. This essay does not aim to systematically review the copious mammalian polyP literature. Instead, we examined polyP synthesis and functions in various microorganisms and used an evolutionary perspective to theorise key issues of this field and propose solutions. By highlighting the presence of VTC4 in distinct species of very divergent eucaryote clades (Opisthokonta, Viridiplantae, Discoba, and the SAR), we propose that whilst polyP synthesising machinery was present in the ancestral eukaryote, most lineages subsequently lost it during evolution. The analysis of the bacteria-acquired amoeba PPK1 and its unique polyP physiology suggests that eukaryote cells must have developed mechanisms to limit cytosolic polyP accumulation. We reviewed the literature on polyP in the mitochondria from the perspective of its endosymbiotic origin from bacteria, highlighting how mitochondria could possess a polyP physiology reminiscent of their ‘bacterial’ beginning that is not yet investigated. Finally, we emphasised the similarities that the anionic polyP shares with the better-understood negatively charged polymers DNA and RNA, postulating that the nucleus offers an ideal environment where polyP physiology might thrive.

## Introduction

Inorganic polyphosphate (polyP) is defined as a chain of phosphate residues linked by high-energy phosphoanhydride bonds (PO₄³^−^, hereafter simplified as Pi). Chain lengths vary widely, from three phosphate residues (excluding pyrophosphate) to several thousand, and they can be found in many different species and under different growth conditions performing diverse functions (we recommend the interested readers the following reviews [1–3]. Research into polyP began in the early 19th century, initially focusing on polyP in microorganisms where the polymer is abundant. In the 20th century, Igor Kulaev and Arthur Kornberg made several significant discoveries that formed a basic understanding of the enzymes involved in polyP synthesis, an important milestone in the history of interdisciplinary chemistry and biochemistry [4,5]. This work enabled many subsequent researchers to further study this polymer which ultimately led to the discovery of polyP in many model organisms across the tree of life. The combined endeavours of these researchers have not only enhanced our comprehension of polyP biosynthesis mechanisms but have also expanded our knowledge of the diverse functions that polyP serves in different species.

The first insights into polyP biology in mammals were reported in the 1960s [6,7]. However, it is relatively recently from the 1990s [8] that mammalian polyP research generated excitement, linking this molecule with several biological processes. Prior to embarking on an evolutionary evaluation of these new findings, it is necessary to highlight two prominent challenges that complicate the study of polyP in mammalian cells. First, polyP levels are significantly lower in mammalian cells than bacteria or yeast [8,9]. As a result, accurate and robust measurements of polyP in mammalian cells demand highly sensitive and reliable analytical techniques. The limited availability of standardised polyP techniques has led to challenges in replicating many of the findings documented in literature. Second, the lack of knowledge of the biosynthetic mechanisms of polyP in mammalian cells has greatly hampered meaningful investigations into the physiological roles it may play. Despite these difficulties, polyP has been shown to play regulatory roles in various cellular processes such as cell growth, differentiation, apoptosis, and immune responses [10–12]. It has also been seen to interact with proteins, nucleic acids and other cellular components, modulating their functions and influencing signal transduction pathways [13–15]. Moreover, polyP has been associated with a wide range of physiological functions in mammals such as blood clotting, bone mineralisation, vascular homeostasis and energy metabolism [12,16,17]. Dysregulation in the metabolism of polyP can also be observed alongside several pathological conditions including thrombosis, inflammation, and neurodegenerative disorders such as Alzheimer's disease [15,18,19].

Due to the technical challenges in measuring polyP levels and the lack of knowledge of polyP synthesis, those involved in mammalian polyP studies mostly resorted to two approaches to study this polymer. The first relies on the chemical-physical nature of polyP as a negatively charged polymer. The other approach is to examine the known roles of polyP in bacteria or single-cell eukaryotes and attempt to determine whether there are equivalencies in mammals. So far, the bulk of mammalian polyP studies have focussed on its role in the mitochondria or in the blood coagulation cascade meaning its potential role in other fundamental areas of biology and disease remains largely unexamined. In this assay, we aim to review the synthesis, chemical properties and functions of polyP in microorganisms and use an evolutionary perspective to theoretically address some of the questions surrounding polyP in mammalians. Within, we suggest that a highly negatively charged molecule such as polyP, is potentially a key player in many fundamental aspects of mammalian biology and it is thus worthy of continuous exploration beyond mitochondrial activity and blood coagulation.

## VTC-complex phylogenetic distribution and its vesicular association

In fungi and several protists such as *Chlamydomonas*, *Trypanosoma*, and *Leishmania*, polyP is synthesised by a membrane-associated protein complex named the vacuolar transporter chaperone (VTC) complex [15,20]. The VTC complex synthesises polyP from ATP on the cytosolic side of vesicular organelles and translocates it into the luminal side, a process requiring the proton gradient generated by the V-ATPase [15]. In budding yeast *Saccharomyces cerevisiae*, the VTC complex consists of four subunits: Vtc1, the mutually exclusive Vtc2 or Vtc3, the catalytic subunit Vtc4 and Vtc5. While Vtc4 is the enzymatic subunit, all four subunits are required for polyP accumulation [20]. Innovative cryo-electron microscopy studies have revealed that the transmembrane domains of all four VTC-complex subunits integrate into the vesicular membrane by forming a channel-like structure. It has been hypothesised that newly synthesised polyP translocates into the vesicular lumen through this channel [21,22]. As a result, polyP is compartmentalised in acidocalcisomes or acidocalcisome-like organelles such as the yeast vacuole or mammalian lysosome.

**Figure 1.**
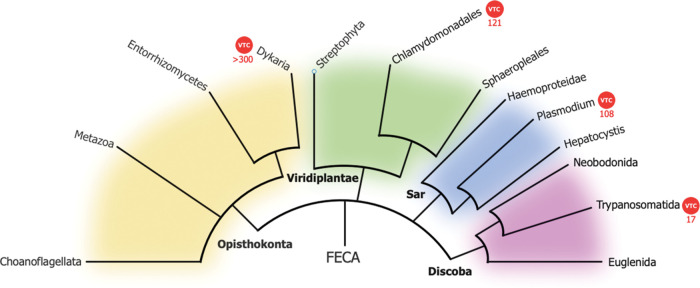
Phylogenetic tree showing the presence of the VTC domain containing proteins. This phylogenetic tree simplified chart was constructed using as reference the Lifemap NCBI version (https://lifemap-ncbi.univ-lyon1.fr). Our analysis of VTC domain distribution across the eukaryotic spectrum shows that the evolutionary presence of the VTC protein (indicated by red circle) is present in the genomes of widely diverse species. Each supergroup is colour-coded for easy identification (Opisthokonta in yellow, Viridiplantae in green, Sar in blue and Discoba in pink). The red number represent the number of reference eukaryote proteomes possessing VTC domain containing protein. These numbers were obtained by screening the InterPro database (https://www.ebi.ac.uk/interpro/) accessed in July 2023 using the VTC domain signature (IPR018966).

Our analysis of the distribution of Vtc4 throughout the eukaryotic spectrum (Figure 1) reveals that Vtc4 is encoded in the genomes of highly divergent species. It is found in Dikarya, a group that includes yeast and fungi and belongs to the Opisthokonta clade. This clade includes all the metazoan however within Opisthokonta, Vtc4 is only present in fungi which means it is not present in mammals. Vtc4 is also largely absent from the plant clade Viridiplantae although it is present in the Chlamydomonadales order of microalgae. Additionally, Vtc4 is present in Trypanosomatida belonging to the Discoba clade, in Plasmodium species that are Alveolates and classified within the variegated SAR (Stramenopiles, Alveolates, and Rhizaria) supergroup. These very divergent eukaryotes clades (Opisthokonta, Viridiplantae, Discoba) and the SAR supergroup separated early during the evolution of eukaryotes, probably soon after the formation of the first eukaryotic common ancestor (FECA). It is therefore highly likely that Vtc4 was encoded by the FECA genome but was then lost from most of the eukaryotic lineages through subsequent evolution. What are the characteristics shared by yeast, Chlamydomonas, Trypanosoma and Plasmodium that led to these genomes retaining Vtc4 to synthesise mainly vesicular-enclosed polyP? Conversely, why was Vtc4 lost from most eukaryote lineages including metazoan/mammals? Given that polyP accumulates in a vesicular compartment such as the human platelet dense granule [23], why have mammals lost the VTC-complex which so efficiently synthesises and transfers polyP through membrane? These are experimentally difficult questions to address as we do not yet fully understand the biology of polyP in these highly divergent organisms. In the meantime, speculation from an evolutionary perspective might help shine a light.

We start by looking at VTC4's vesicular association. Vesicles play crucial roles in transporting molecules, sorting cargo, and maintaining cellular homeostasis [24]. The compartmentalisation of polyP in vacuoles or acidocalcisomes allows it to accumulate while preventing undesired increases in cytosolic polyP levels. This is of vital importance as excessive cytosolic polyP could sequester essential cations and potentially interact with positively charged proteins. Indeed, outside of the vacuole overexpression of bacterial polyphosphate kinase PPK1 is toxic to the yeast [25,26]. Additionally, the vesicular compartments contain specialised enzymes and transporters that facilitate the breakdown of polyP which in turn controls the release of Pi into the cytoplasm. The yeast *S. cerevisiae* is the experimental organism that currently offers the most insight into polyP catabolism. *S. cerevisiae* encodes four enzymes responsible for polyP degradation: the cytosolic exopolyphosphatase Ppx1, the cytosolic endopolyphosphatase Ddp1 and the vacuolar endopolyphosphatases Ppn1 and Ppn2 [15,27]. Organisms such as yeast and Chlamydomonas live in environments where Pi levels fluctuate. Vesicular production and storage of polyP allow them to accumulate polyP when Pi is abundant and to break it down into Pi for essential cellular metabolisms when Pi is scarce. Why parasites *Plasmodium*, *Trypanosoma*, and *Leishmania* encode the VTC complex is less obvious and a potential answer requires detailed consideration of their different life stages. Pi concentration is extremely stable in mammals, as mammalian Pi homeostasis is strictly controlled by the three hormones fibroblast growth factor-23 (FGF-23), calcitriol, and parathormone (PTH) [28]. These hormones modulate intestinal Pi uptake, renal Pi excretion and reabsorption, and the absorption or release of Pi by bones [29]. It is therefore unlikely that polyP is required for buffering Pi while parasites live in human hosts. *Plasmodium*, *Trypanosoma*, and *Leishmania* have insects as an intermediary host. However how Pi homeostasis is controlled in insect, if at all, remains elusive. Their exoskeleton — which provides structural support to their organism, as bones do in higher animals — is made of long-chain polymers of N-acetylglucosamine instead of calcium-phosphate deposits of hydroxyapatite. This means that exoskeletons cannot function as Pi reservoirs as bones do. A recent study showed that in Drosophila, Pi homeostasis is controlled through specialised gut cells which use a special membranous organelle known as PXo bodies which carry a Pi-sensitive XPR1 Orthologue (PXo) [30]. The peculiarity of Pi homeostatic mechanisms in insects is likely to mean that inside the insect body Pi levels fluctuate widely. These fluctuations would in turn suggest that retaining the VTC complex — which enables the storage of free Pi as polyP — is a critical necessity for parasites such as *Plasmodium, Trypanosoma*, and *Leishmania*, so they can have a steady source of polyP during the lifecycle period where their host is an insect.

**Figure 2.**
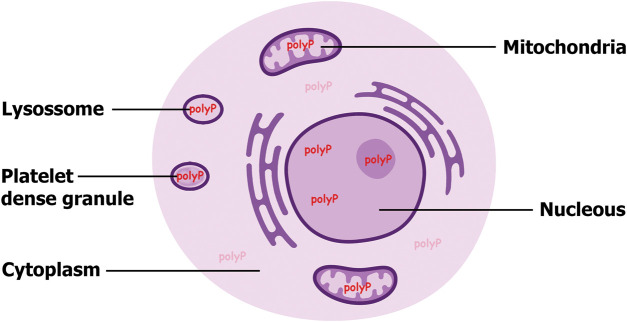
Intracellular distribution of polyphosphate (polyP) within a mammalian cell. It is likely that polyP (red) is not restricted to a singular organelle. To regulate the blood coagulation cascade, polyP must accumulate in a vesicle compartment such lysosome and the platelet specific dense granules. Additionally, evidence supports polyP presence within the mitochondria, and emerging studies suggest its presence within the nucleus. Consequently, the cytosol of mammalian cells is likely to maintain non-toxic levels of polyP (pale red), at the very least, to transit between synthesis site and the various compartments where polyP tends to accumulate.

VTC4's involvement in the vesicular accumulation of polyP may have provided selective advantages throughout the evolution of lineages facing high levels of Pi fluctuation. Although cordate/mammalian lineages have lost the VTC complex — potentially because of the tightly regulated Pi homeostasis previously discussed — they still generate polyP and store it in vesicles the best known are platelet dense granules (Figure 2). We speculate that mammals evolved a different multiprotein complex that substitutes the VTC complex function. Like the VTC complex, this hypothetical multiprotein complex could couple synthesis and membrane translocation or alternatively, it could transport/translocate polyP synthesised in the cytoplasm to the vesicles. In either scenario, the membrane translocation of polyP, a long and highly charged polymer, is likely to require the formation of a membrane-associated channel like the one formed by the VTC subunits. Numerous proteomic studies have been performed on platelets and on the platelet dense granules [31–34]. It is thus likely that these proteins have already been identified but have not yet been associated with the synthesis and/or translocation of polyP. Careful re-analysis of this proteomic data could therefore be instrumental to our understanding of polyP biology in mammalian cells.

## Bacterial polyP synthesis by PPK1, its toxicity and secretion in amoebae

The main enzyme involved in the metabolism of polyP in bacteria is the polyphosphate kinase 1 (PPK1) which reversibly catalyses the polymerisation of the gamma-phosphate in ATP into the polyP chain [35]. Some bacteria encode a second enzyme, PPK2, which synthesises polyP *in vitro* but carries out the reverse reaction *in vivo,* where polyP is used to phosphorylate nucleoside diphosphates [36]. PolyP is synthesised by PPK1 either during the stationary phase of bacterial growth or under stress conditions such as nutrient limitation and oxidative stress [37,38]. PPK1 is absent from all eukaryotes except *Dictyosteliida* and interestingly, as mentioned earlier, the cytosolic expression of *E. coli* PPK1 has to led to toxicity in yeast [25] and mammalian cell alike [9]. The social amoeba *Dictyostelium discoideum* encodes a homologue of the bacterial PPK1 gene named DdPPK1 [15,39]. These amoeba feed on bacteria and they thus acquire PPK1 through horizontal gene transfer rather than through having a direct shared ancestry [15,36]. Vegetative logarithmic growing *D. discoideum* possesses far lower polyP than budding yeast [40] and intracellular levels of polyP increase during starvation induced development [39]. Unique to *D. discoideum* however is its ability to accumulate extracellular polyP likely secreted by the amoeba itself. This allows the amoeba to sense the local cell density and it may enable it to anticipate when it will overgrow its food source [41]. The exact function played by the secreted polyP is understudied, however it has been proposed that extracellular polyP binds to G protein-coupled polyP receptor (GrlD) [42]. Conversely, during starvation induced development, a significant portion of an amoeba's ATP is dedicated to the synthesis of polyP which is then secreted. This higher energy expenditure indicates that polyP is crucial to the function of the amoeba. DdPPK1 does not contain any transmembrane or intraorganellar localisation signals and therefore the synthesis of polyP in the amoeba occurs in the cytosol. The toxicity of high levels of cytosolic polyP is most likely circumvented by its efficient secretion, however the secretory mechanisms have not yet been characterised. We speculate that this secretion of polyP may be achieved using a plasma-membrane associated channel or, alternatively, through vesicular intermediates that carry a polyP membrane translocation channel in similar fashion to the structure that must exist on the membrane of platelet dense granules.

Thus far our analysis of the two characterised eukaryotic polyP synthetic mechanisms, the VTC-complex and the amoeba's DdPPK1, has highlighted how eukaryote cells must possess a dedicated organelle to accumulate large amounts of polyP or have a polyP secretory pathway, both of which are likely to have evolved to prevent polyP toxicity in the cytosol.

## Mitochondria polyP: more than a plain phosphate/energy storage molecule

The defining event in the evolution of eukaryotes was the acquisition of a bacterial endosymbiont that gave origin to the mitochondria [43,44]. It is reasonable to imagine that the endosymbiont alpha-proteobacterium was carrying the PPK1 and/or PPK2 gene. During the early phase of the endosymbiont, the PPKs’ coding sequence was not retained by the FECA. PolyP metabolism is prevalent in bacteria [45] therefore its presence in today's mitochondria could be related to its bacterial origin. These considerations suggest first, that mitochondria may have acquired the ability to import polyP from the cytosol or developed an alternative polyP synthetic pathway to compensate for the loss of PPK1. Second, that mitochondria could possess a polyP physiology reminiscent of their ‘bacterial’ origins. Bacterial polyP's functions in biofilm formation, stringent response and stationary phase survival [46] cannot be found in mitochondria. However, polyP also enables bacterial endurance against many environmental stressors such as metals, changes in pH, osmotic stress, heat, and oxidants [2,47]. Mitochondria play a pivotal role in eukaryotic cell pH regulation through inner membrane proton translocation as well as in maintaining metal homeostasis such as iron [48,49]. Additionally, this organelle is the primary source of redox signalling molecules [49,50]. These functions might have evolved from the similar roles played by polyP in bacteria. Indeed, a recent article linked mitochondrial polyP with the generation of reactive oxygen species (ROS) [51] demonstrating that the function of bacterial polyP in the response to oxidants might have been retained by mitochondria. This reflection should stimulate further investigation on the role of mitochondrial polyP in regulating eukaryote pH and cation homeostasis. It should not come as a surprise however that research into polyP mitochondria is already an active investigative area (Figure 2), mainly due to the intrinsic property of polyP as an energy and Pi reserve [52]. Studies have shown that polyP can influence the ratio at which a cell uses oxidative phosphorylation or glycolysis to generate ATP [53] as well as mitochondrial membrane potential and calcium homeostasis [54], both of which are critical for maintaining mitochondrial integrity and functionality. Through being present in the mitochondria, polyP may directly modulate these processes and contribute to cellular homeostasis as well as energy metabolism overall [55,56].

This said, a direct role of polyP as an energy source is unlikely. Mitochondria are known as the cell's powerhouse, responsible for generating energy in the form of adenosine triphosphate (ATP) through oxidative phosphorylation [49]. PolyP, with its high-energy phosphoanhydride bonds, could serve as a readily available source of energy in a situation where ATP synthesis becomes hampered. However, we should consider that cell metabolism demands an exceptionally rapid turnover of ATP. Therefore, based on the concentrations of polyP reported in literature, polyP as energy donor would be able to sustain life for a fraction of a second in the case of mammalian cells and a few seconds in the case of bacteria [57]. It is therefore unlikely that the high energetic phosphonamidite bonds of the mitochondrial polyP pool meaningfully function as chemical energy storage.

PolyP presence in mitochondria might instead be involved in the Pi storage process of this organelle. The stored polyP can be rapidly hydrolysed, releasing Pi groups utilised by the ATP-synthase to produce ATP. This localised pool of polyP can serve as a dynamic and efficient Pi buffer, perhaps regulated independently from the tightly regulated mitochondrial Pi importer mechanism [58,59]. Mitochondrial polyP could enable a rapid response to the energy demands of the cell by providing Pi when the cytosolic Pi supply is reduced. The mitochondrial location of polyP further highlights the importance of compartmentalisation in cellular biochemistry. By sequestering polyP within mitochondria, the cell can regulate this polyP pool independently from other pools (for example polyP vesicular accumulation) and prevent its potentially toxic accumulation in the cytosol. It is extremely unlikely albeit still unknown, that mammalian cells possess three different polyP synthesising mechanisms: one for the vesicle, another for the mitochondria, and a third for the nucleus (see below). Mammalian cell cytosol must possess small non-toxic levels of polyP (Figure 2) which can, as a minimum, be shuttled between the site of synthesis and the different compartments where polyP appears to accumulate.

## The polyanion polyP: exploring its nuclear localisation

One important aspect of polyP is its polyanionic nature. The negative charges of the more famed nuclear polymers, the nucleic acids DNA and RNA, have significant implications for chromatin packaging, gene expression regulation, RNA processing and nuclear transport. The electrostatic interactions between negatively and positively charged molecules such as histones are vital for various processes and contribute to the overall functionality of the nucleus. PolyP, whilst not as famous as DNA and RNA, also exhibits a negatively charged polymeric structure with a high degree of flexibility, enabling it to encapsulate positive charged proteins [60]. The nuclear environment which adapted to store, metabolise and process the anionic DNA and RNA could therefore also be the ideal environment where the physiology of polyP can prosper. The presence of polyP within the nucleus of mammalian cells is still being explored (Figure 2), however several recent studies discussed henceforth have provided emerging evidence supporting its presence and potential functional roles within the nucleus.

The Ruiz lab [61] described that intracellular polyP in myeloma cells — a malignant transformation of plasma cells — is mostly accumulated within the nucleolus and exhibits co-localisation with nucleolar markers through confocal microscopy. The authors suggest that the presence of polyP within the nucleolus could be dependent on ribosome subunit synthesis given that the inhibition of transcription by actinomycin D — a well-known inhibitor of nucleolar RNA synthesis — induces polyP to move from the nucleoli to the cytoplasm in these cells. Cisplatin treatment is known for its ability to induce DNA damage [62] and other cancer cell lines besides myeloma respond to this treatment with a robust up-regulation and cellular redistribution of endogenous polyP which leads to the formation of distinct nucleolar polyP foci [63]. These findings indicate that polyP may play a role in the regulation and/or processing of ribosomal RNA synthesis due to the co-localisation of RNA Pol I and polyP upon cisplatin stress. This data aligns with a study by Bru et al. [64] which suggests that polyP may play a crucial role in the process of DNA damage recovery by acting as a protective factor in supporting the synthesis of triphosphate deoxynucleotides (dNTPs). These are essential for DNA replication, repair, and other DNA-dependent processes [65]. This emerging evidence suggests that nuclear polyP might play a significant role in chromatin dynamics, gene expression and other processes, especially those localised in the nucleolus such as rRNA transcription/processing and/or ribosome assembly.

Interestingly, recent studies have revealed that many nucleolar proteins undergo a unique post-translational modification (PTM) called lysine-polyphosphorylation [66,67]. This modification involves the covalent attachment of polyP chains to lysine (Lys) residues, providing a novel mechanism for protein regulation and function [14,66]. The addition of polyP chains can alter the charge of the target's proteins which can have significant implications for their interactions with other proteins and nucleic acids. This charge modification may also lead to substantial changes in protein conformation and binding affinity. Polyphosphorylation itself has the potential to directly compete with other well-known lysine modifications such as acetylation, methylation, ubiquitylation, SUMOylation, and glycation [68,69]. This competitive relationship between lysine-polyphosphorylation and other modifications could influence the overall functional status of the protein, affecting its stability, location or interaction with other cellular components. Furthermore, lysine residues, along with arginine (Arg) residues, have long been recognised as crucial components of the nucleolar localisation sequence [70], lending further support to the notion that lysine-polyphosphorylation could play an important role in the nucleolus.

It should be noted that the same pool of nucleolar proteins targeted by lysine-polyphosphorylation is also targeted by serine-pyrophosphorylation [71,72] and other non-canonical PTM driven by inositol pyrophosphates (PP-IPs) such as diphosphoinositol pentakisphosphate (IP_7_) and bis-diphosphoinositol tetrakisphosphate (IP_8_) [73,74]. Inositol pyrophosphates regulate rRNA transcription [75,76] and nucleolar architecture [77]. Nucleolar physiology therefore appears to be another research area, besides the PP-IPs regulating polyP synthesis by the yeast VTC complex [78], where these two negatively charged PP-IPs and polyP molecules functionally cross-talk. The mammalian polyP endopolyphosphatase has been recently identified as Nudt3 [79] — a phosphatase also active towards PP-IPs [27,80] — further stressing the functional interaction between these two classes of molecules. Further effort is urgently required to evaluate the cross-talk between lysine-polyphosphorylation and serine-pyrophosphorylation and its physiological significance within the nucleolus in an environment enriched by hyper-phosphorylated proteins. Such an environment appears to welcome the negatively charged polyP and PP-IPs and their presence might contribute to the formation of the nucleolus itself. The phase separation nature of the nucleolus [81,82] could be modulated by enhancing or reducing the charge of nucleolar proteins and thus the nucleolar localisation of polyP, which has been recently shown to induce phase separation [83] similar to DNA and RNA [84], could modulate the nucleolus’ organisational nature. Understanding the precise location and functional significance of polyP within the nucleus is an emerging area of investigation and further research is needed to establish the precise mechanisms and functional implications of this polymer within nuclear/nucleolus.

Could the polyP polymer mirror the nucleus's ultimate function and contain biological information? The recent discovery that ultra-phosphates, branched polyP chains, could exist in water [85] theoretically permits the existence in cells of branched polyP. Diverse branching patterns might generate different polyP carrying diverse functions. It might appear too far-fetched to hypothesise that polyP, a single-letter ‘thought linear’ polymer, could hold some sort of code/information. However, original discoveries and innovative ideas, that are the foundation of breakthroughs, are needed for the young polyP research field to mature. With the current essay, we hope to have stimulated useful thoughts to propel the already thrilling polyP research to an even more exciting future.

## Perspectives

- The past 20 years have witnessed a steady growth in mammalian polyP interest. Unquestionably, polyP is associated with a wide range of physiological functions and pathological states.
- Not knowing how the different pools (Figure 2) of polyP are synthesised and metabolically interconnected is hampering decisive progress. The unique evolutionary perspectives presented in this assay might guide future research.
- The polyP research field requires the development of reliable detection methods to allow the full characterisation of mammalian polyP enzymology. Thinking outside the box might also benefit the still-young mammalian polyP research fields.

## Open Access

Open access for this article was enabled by the participation of University College London in an all-inclusive *Read & Publish* agreement with Portland Press and the Biochemical Society under a transformative agreement with JISC.

## Abbreviations

FECA: first eukaryotic common ancestor
FGF-23: fibroblast growth factor-23
IP7: diphosphoinositol pentakisphosphate
IP8: bis-diphosphoinositol tetrakisphosphate
polyP: inorganic polyphosphate
PP-IPs: inositol pyrophosphate
PPK1: Polyphosphate kinase 1
PTH: parathormone
PTM: post-translational modification
PXo: Pi-sensitive XPR1 orthologue
ROS: reactive oxygen species
rRNA: ribosomal RNA
SAR: Stramenopiles, Alveolates, and Rhizaria supergroup
VTC: vacuolar transport chaperone
